# Comparative outcomes of Hugo™ robotic and laparoscopic sacrocolpopexy in a high-volume tertiary center: a propensity-matched study

**DOI:** 10.1007/s11701-025-02915-z

**Published:** 2025-11-01

**Authors:** S. Mastrovito, Davide Arrigo, C. Riccetti, G. Campagna, F. Natale, A. Ercoli, A. Fagotti, F. Fanfani, G. Panico

**Affiliations:** 1 Department of Woman, Child and Public Health, Gynecologic Oncology Complex Unit, Fondazione Policlinico Universitario A. Gemelli IRCCS, Rome, 00168 Italy; 2 Department of Woman, Child and Public Health, Gynecologic Oncology Complex Unit, Catholic University of the Sacred Heart, Fondazione Policlinico Universitario A. Gemelli IRCCS, Largo Agostino Gemelli 8, Rome, Italy; 3 Complex Operating Unit of Surgical Gynecology and Urogynecology, Isola Tiberina - Gemelli Isola Hospital, Rome, Italy; 4 Unit of Gynecology and Obstetrics, Department of Human Pathology of Adults and Developmental Age, University Hospital G. Martino, Messina, 98100 Italy

**Keywords:** Hugo RAS system, Sacrocolpopexy, Pelvic organ prolapse, Robotic surgery, Urogynecology

## Abstract

**Objective:**

Minimally invasive sacrocolpopexy (SCP) is regarded as the gold standard procedure for treating Pelvic Organ Prolapse (POP). Robotic-assisted surgery has emerged as a solid alternative to laparoscopy. Recently, novel platforms have been introduced, including the Hugo RAS (MEDTRONIC Inc, USA), a modular system featuring independent bedside units and an open-designed console. Although solid evidence has shown comparable outcomes between RAS and laparoscopy, there is still limited evidence on the safety and feasibility of novel multi-arm robotic platforms.

**Design:**

We conducted a retrospective study analyzing data from 450 patients undergoing minimally invasive SCP (May 2022-December 2023), using propensity score matching to correct for treatment selection bias. The primary aim was to assess differences in 30-day Clavien-Dindo grade ≥ II complications, Secondary outcomes included intraoperative complications, estimated blood loss, operative time, length of hospital stay, and functional outcomes, to assess overall safety, feasibility, and outcomes of laparoscopic versus robotic-assisted SCP performed with the Hugo RAS system.

**Setting:**

All patients underwent minimally invasive SCP at Fondazione Policlinico Universitario Agostino Gemelli IRCCS, Rome, Italy.

**Participants:**

After excluding patients with missing data or incomplete follow-up and PSM, 284 patients were compared, 142 who underwent laparoscopic SCP and 142 who underwent robotic SCP with the Hugo™ RAS system.

**Intervention:**

The study aims to compare laparoscopic and robotic SCP performed with the Hugo™ RAS platform.

**Results:**

Short- to mid-term outcomes were compared. RAS did not significantly differ from laparoscopy in terms of intra- or post-operative complications and length of hospitalization. The robotic group showed a slightly longer operative time and slightly lower intraoperative blood loss. The mean follow-up was 18 months (range 12–36) and a statistically significant improvement of objective and subjective outcomes was reported in both populations, with high satisfaction rate.

**Conclusions:**

The Hugo™ RAS system resulted safe and effective to perform SCP for symptomatic POP, representing a feasible alternative to laparoscopy. These findings support its use as an alternative for the treatment of symptomatic POP and contribute to expanding the evidence for newer robotic platforms in urogynecologic and reconstructive pelvic surgery.

## Introduction

Pelvic organ prolapse is a prevalent condition that significantly impacts women’s quality of life, necessitating surgical intervention in most cases [1, 2]. Minimally invasive sacrocolpopexy is the current gold standard for POP treatment, offering superior anatomical and functional outcomes over native tissue repair, with a better perioperative safety profile when compared to open surgery [1, 3, 4]. However, conventional laparoscopic SCP presents technical challenges, derived by the need for deep dissection and for advanced suturing skills to fix the mesh to surrounding tissue which can impact surgical proficiency [5, 6].

Robotic-assisted surgery has emerged as an alternative to standard laparoscopy, addressing some of these challenges by enhancing visualization, precision, and ergonomics [2, 4, 7, 8]. The DaVinci^®^ system has been the dominant RAS platform, yet newer multi-arm robotic systems, such as the Hugo RAS system (Medtronic Inc., USA), have been introduced, featuring independent bedside units, wristed instruments, and an open-console design [9, 10].

Despite evidence supporting the comparability of RAS to laparoscopy in SCP, prior studies have primarily focused on DaVinci-based SCP, leaving a gap in knowledge regarding alternative robotic systems [7, 8]. Evidence on the safety and feasibility of SCP using newer multi-arm robotic platforms, such as Hugo RAS, is yet to be defined.

This study aims to address this gap by evaluating and comparing the perioperative and clinical outcomes of SCP performed via laparoscopy versus the Hugo RAS system. Using a large retrospective dataset and propensity score matching (PSM), we seek to provide valuable insights on the safety, feasibility, and potential advantages of this emerging robotic platform in urogynecological surgery.

## Methods

A single-centre observational retrospective and comparative cohort study was conducted on prospectively collected data.

Data from all consecutive patients undergoing minimally invasive SCP for symptomatic POP at “Fondazione Policlinico Universitario Agostino Gemelli IRCCS” between May 2022 and December 2023 were analyzed. Institutional review board approval was obtained before data collection and analysis (no. 0012761/22, approved on 1/2/2022). Written informed consent for the use of anonymized data was obtained from all participants before inclusion in the study.

Patients were included if they underwent either laparoscopic or robotic-assisted SCP using the Hugo RAS system during the study period. The choice of approach was determined solely by the availability of the robotic system, which was accessible once a week, whereas laparoscopic procedures were performed daily. Patients with missing data and those with incomplete follow-up were excluded from the analysis.

Surgical technique was standardized across both approaches, as previously described by our group [5, 6, 9, 10]. All procedures were performed by two experienced surgeons extensively trained in both laparoscopic and robotic surgery. All procedures used lightweight polypropylene mesh (Restorelle XL^®^, Coloplast, USA), with fixation performed by non-absorbable sutures (Ethibond^®^) at both sacral promontory and on the vaginal walls. Patients with an intact uterus underwent concomitant subtotal hysterectomy and salpingectomy. Postmenopausal patients were counselled regarding bilateral salpingo-oophorectomy. The presence of a concomitant hysterectomy was included as a covariate in the propensity-score model to adjust for potential confounding related to surgical complexity.

Patients who underwent other concomitant procedures including anterior and posterior colporrhaphy, anti-incontinence procedures, and ventral rectopexy were excluded from the analysis to avoid additional confounding factors related to surgical complexity.

Perioperative management was uniform throughout the study period, including preoperative assessment, anaesthesia protocol, and postoperative care. Antibiotic prophylaxis consisted of a single perioperative dose of cefazolin 2 g in non-allergic patients. Standardized discharge criteria were applied to all patients.

Demographic, clinical, intraoperative, and postoperative data of 450 patients were collected from the institutional database. Variables were defined according to the International Continence Society classification and the Clavien-Dindo grading system for surgical complications [1, 11, 12]. Early postoperative complications were defined as those occurring within 30 days. Events beyond this window were not included in the primary analysis.

The primary outcome was the occurrence of Clavien–Dindo grade ≥ II complications within 30 days after surgery. Secondary outcomes included intraoperative complications, estimated blood loss, operative time, length of hospital stay, and conversion to open surgery, objective and subjective cure rate. Anatomical (objective) cure rate was defined as postoperative POP-Q stage < 2. Subjective cure was defined as no prolapse symptoms after surgery [11].

Descriptive statistics were used to summarize demographic and clinical characteristics of the study population. Continuous variables were expressed as mean ± standard deviation [6] or median and interquartile range (IQR), depending on data distribution. Categorical variables were expressed as frequencies and percentages. Comparisons between groups were performed using Student’s t-test for normally distributed continuous variables and the Mann-Whitney U test for non-normally distributed variables. Categorical variables were compared using Pearson’s chi-square test or Fisher’s exact test, as appropriate. A two-sided p-value < 0.05 was considered statistically significant.

To reduce selection bias, a PSM analysis was performed using a 1:1 nearest neighbour matching without replacement with a caliper of 0.2 of the standard deviation of the logit of the propensity score. The propensity score was derived from a logistic regression model including clinically and statistically relevant covariates: The propensity score was derived from a logistic regression model including age, BMI, previous abdominal surgeries, previous prolapse repair, previous hysterectomy, preoperative POP-Q stage, and stress urinary incontinence. Covariate balance was evaluated using standardized mean differences and variance-ratio diagnostics. Ratios between 0.5 and 2.0 were considered acceptable. The final matched sample included 142 patients per group. Matched groups were compared using the Wilcoxon signed-rank test for continuous variables and the McNemar test for categorical variables. As a sensitivity analysis, inverse-probability-of-treatment weighting (IPTW) with stabilized weights was performed using the same covariates included in the propensity-score model. Covariate balance was reassessed, and weighted regression models were used to estimate treatment effects.

All statistical analyses were performed using SPSS v29.0.13 [13].

## Results

**Fig. 1:**
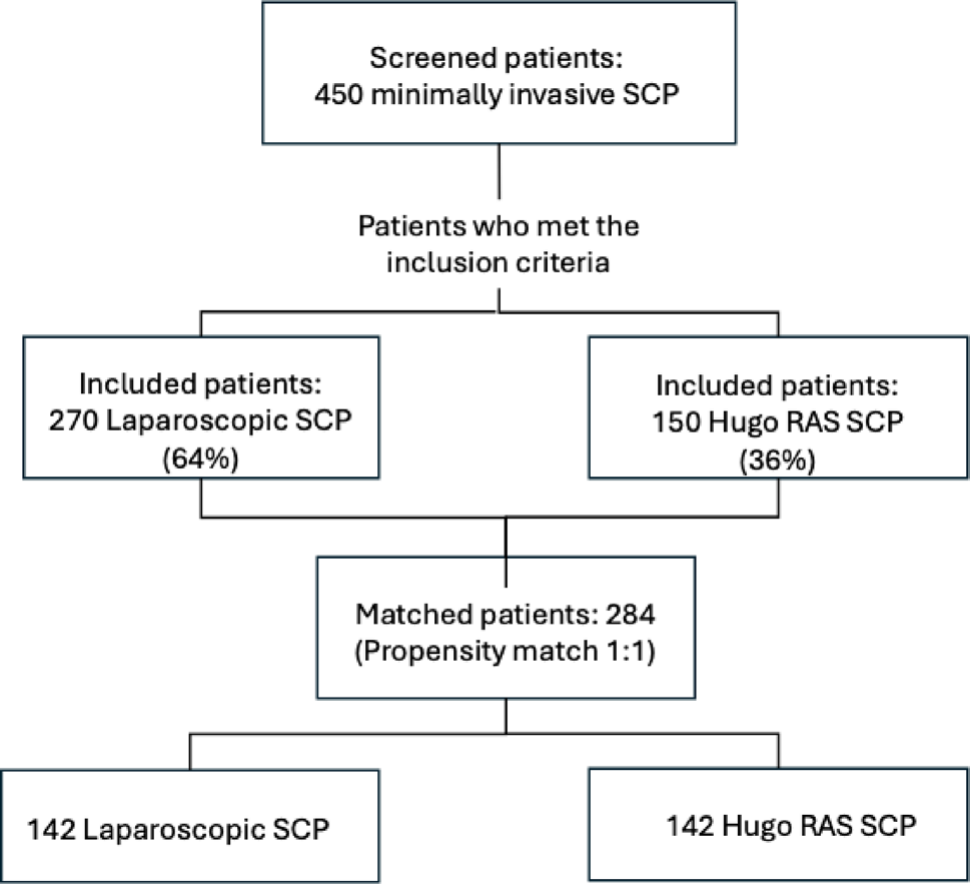
Flow-diagram of patient selection process

After matching, 284 patients remained in the analysis, with 142 undergoing laparoscopic SCP and 142 undergoing robotic-assisted SCP (Fig. 1).

Table 1 shows patients’ baseline characteristics of the unmatched and matched population, grouped according to the employed surgical approach.

**Table 1:** Patient demographics. Patients’ baseline characteristics of the unmatched and matched population

Variable	Unmatched population			Matched population		
	LPS (*n* = 270)	Hugo RAS (*n* = 150)	*p*-value	LPS (*n* = 142)	Hugo RAS (*n* = 142)	*p*-value
Age (years), median (range)	65 (44–84)	69 (37–84)	0.12	65 (44–77)	70 (37–84)	0.21
BMI (kg/m2), median (range)	24 (19–33)	25 (19–34)	0.58	25 (19–33)	25 (19–34)	0.76
Parity, median (range)	2 (0–5)	2 (1–5)	0.15	2 (1–5)	2 (1–5)	0.42
Previous abdominal surgery, N(%)	95 (35.2)	60 (40)	0.33	50 (35.3)	57 (39.9)	0.42
Hysterectomized, N (%)	44 (16.3)	22 (14.7)	0.76	21 (14.8)	22 (15.4)	0.77
Preoperative POP-Q stage	3 (3–4)	3 (2–4)	0.29	3 (3–4)	3 (3–4)	0.87
Anterior POP-Q stage, median (range)	3 (1–4)	3 (1–4)	0.37	3 (1–4)	3 (1–4)	0.56
Apical POP-Q stage, median (range)	3 (2–4)	3 (2–4)	0.15	3 (2–4)	3 (2–4)	0.51
Posterior POP-Q stage, median (range)	1(0–4)	1(0–4)	0.34	1(0–4)	1(0–4)	0.60
Preoperative SUI, N (%)	104 (38.5)	53 (35.3)	0.52	50 (35.2)	51 (35.9)	0.94

**Fig. 2:**
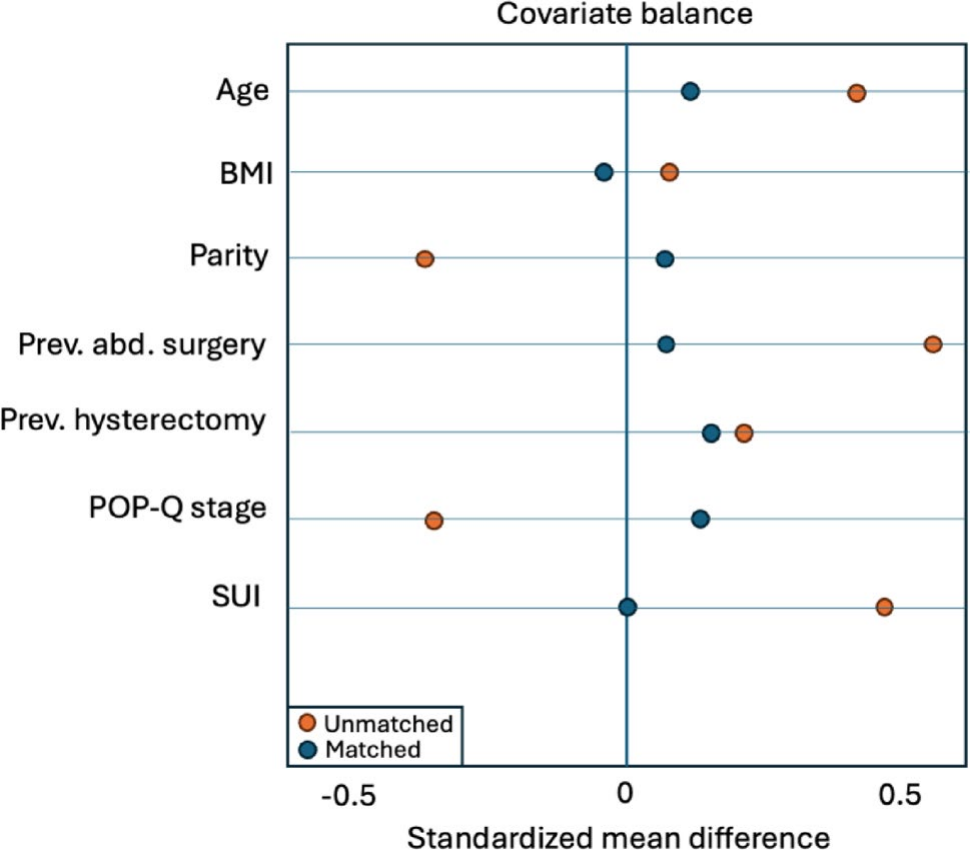
Love plot diagram depicting standardized mean differences before and after matching

No significant preoperative differences were found between the laparoscopic and robotic group in term of age, BMI, parity, previous abdominal surgery, % of previous hysterectomy, preoperative POP-Q stage, presence of stress urinary incontinence. Figure 2 shows the effect of the PSM process on the standardized mean difference. The PSM demonstrated a satisfactory balance between the laparoscopic and Hugo RAS groups, as shown by the standardized mean differences < 0.1. After IPTW, baseline covariates were well balanced (all standardized mean differences < 0.1). Weighted analyses confirmed no significant differences between Hugo RAS and LPS in the primary endpoint.

Table 2 shows intra- and postoperative results for both the unmatched and matched populations. A significant difference was observed in mean operative time, with longer required time for the Hugo RAS group in both the unmatched (139.8 ± 38.0 vs. 153.2 ± 32.6, *p* <.001) and the matched populations (140.8 ± 37.3 vs. 153.7 ± 32.6, *p* =.002). No significant difference was found in estimated blood loss before matching, but a slight difference was evidenced by the matching process in favour of robotic approach (63.6 ± 85.7 vs. 38.9 ± 32.1 (Hugo RAS), *p* =.002).

**Table 2:** Peri-operative data. Intra- and postoperative results for both the unmatched and matched populations

Variable	Unmatched population			Matched population		
	LPS (*n* = 270)	Hugo RAS (*n* = 150)	*p*-value	LPS (*n* = 142)	Hugo RAS (*n* = 142)	*p*-value
OT (min), median (range)	135 (49–250)	150 (87–260)	**< 0.001**	135 (49–249)	150 (87–260)	**0.002**
EBL (mL), median (range)	20 (0–500)	30 (10–150)	0.12	30 (0–500)	30 (10–150)	**0.002**
Time to discharge (days), median (range)	3 (2–4)	3 (2–5)	0.87	3 (2–4)	3 (2–5)	0.95
Intraoperative complications, N (%)	9 (3.3)	8 (5.3)	0.32	6 (4.2)	8 (5.6)	0.60
Postoperative complications, N (%)	10 (3.7)	7 (4.6)	0.12	7 (4.9)	7 (4.9)	1.0
Grade I	6 (60)	2 (29)		4 (57)	2 (29)	
Grade II	3 (30)	5 (71)		3 (43)	5 (71)	
Grade IIIa	0	0		0	0	
Grade IIIb	1 (10)	0		0	0	

There were no conversions either from robotic to laparoscopic or open approach in both groups. Time to discharge was superimposable between groups, both before and after matching. In our cohort, robotic-assisted surgery with the Hugo RAS system did not significantly differ from standard laparoscopic procedure in terms of intra- and post-operative complications and length of hospitalization. Intraoperative complications rate was comparable (4.2% vs. 5.6%, *p* =.6) in the laparoscopic group and Hugo™ RAS group, respectively.

Early postoperative complications rate was superimposable between the two groups after matching (4.9% for both groups). One major complication was recorded in the laparoscopic group (grade IIIB), specifically a post-operative bleeding in the presacral area requiring surgical drainage of a retroperitoneal hematoma. Nevertheless, the case was excluded after matching.

No cases of mesh-related complications were reported in both groups, and no re-operations requiring mesh removal or excision were needed.

The mean follow-up was 18 months (range 12–36) and a statistically significant improvement of objective and subjective outcomes was reported in both populations. There was a significant POP symptoms resolution and no difference between groups in term of satisfaction rate, measured as Patient Global Impression of Improvement (PGI-I) ≤ 2. All recurrences were minor (POP-Q ≤ stage II) and did not require reoperation. (Table 3).

**Table 3:** Postoperative outcomes. Postoperative outcomes for both the unmatched and matched populations

Unmatched population								
Variable	LPS (*n* = 270)			Hugo RAS (*n* = 150)			*p*-value	
Anatomical cure rate, n (%)	251 (93)			142 (94.7)			0.5	
Subjective cure rate, n (%)	260 (96.3)			147 (98)			0.33	
Stress urinary incontinence, n (%)	104 (38.5)			53 (35.3)			0.162	
PGI-I, n (%)							0.39	
1–2	264 (97.4)			148 (98.7)				
> 2	7 (2.6)			2 (1.3)				
	**Pre-op**	**Post-op**	**p-value**	**Pre-op**	**Post-op**	**p-value**		
POP-Q stage								
Anterior POP-Q stage, median (range)	3 (1–4)	1 (0–3)	**< 0.001**	3 (1–4)	1 (0–3)	**< 0.001**	0.13	
Apical POP-Q stage, median (range)	3 (2–4)	0 (0–2)	**< 0.001**	3 (2–4)	1 (0–2)	**< 0.001**	0.29	
Posterior POP-Q stage, median (range)	1 (0–4)	0 (0–1)	**< 0.001**	1 (0–4)	0 (0–1)	**< 0.001**	0.76	
**Matched population**								
**Variable**	**LPS (n = 142)**			**Hugo RAS (n = 142)**			**p-value**	
Anatomical cure rate, n (%)	132 (93)			135 (95.1)			0.44	
Subjective cure rate, n (%)	138 (97.2)			139 (97.9)			0.7	
Stress urinary incontinence, n (%)	104 (38.5)			53 (37.3)			0.162	
PGI-I, n (%)							0.4	
1–2	138 (97.2)			140 (98.5)				
> 2	4 (2.8)			2 (1.4)				
	**Pre-op**	**Post-op**	**p-value**	**Pre-op**	**Post-op**	**p-value**		
POP-Q stage								
Anterior POP-Q stage, median (range)	3 (1–4)	1 (0–3)	**< 0.001**	3 (1–4)	1 (0–3)	**< 0.001**	0.13	
Apical POP-Q stage, median (range)	3 (2–4)	0 (0–2)	**< 0.001**	3 (2–4)	1 (0–2)	**< 0.001**	0.29	
Posterior POP-Q stage, median (range)	1 (0–4)	0 (0–1)	**< 0.001**	1 (0–4)	0 (0–1)	**< 0.001**	0.76	

## Discussion

This study aimed to evaluate safety, feasibility, and clinical outcomes of SCP performed using the Hugo™ RAS system compared to conventional laparoscopy in a large, propensity score matched cohort. Our findings demonstrate that both approaches offer similar perioperative safety profiles, with comparable rates of intra- and early postoperative complications, low conversion rates, equivalent length of hospital stay and superimposable functional outcomes at follow up. These results suggest that SCP using the Hugo™ RAS system is a safe and feasible alternative to the laparoscopic approach in the treatment of POP.

The longer operative time observed in the robotic group is consistent with existing literature on RAS and expands the literature in the context of newer platforms. Our findings align with those of prior studies comparing robotic and laparoscopic SCP using the Da Vinci^®^ platform, which consistently demonstrate comparable safety and efficacy of the robotic approach [4, 7, 8, 14–17].

Previous studies using the Da Vinci^®^ system have shown longer operative times compared to laparoscopy, often attributed to docking procedures and learning curve dynamics in the early phases of implementation [18, 19]. The increased operative time in the Hugo group may also partially reflect the institutional learning curve for a newly implemented platform [17, 20]. In our study, the slight difference in operative time did not translate into increased morbidity or prolonged hospitalization, and did not adversely affect patient outcomes. Notably, estimated blood loss was slightly lower in the robotic group after matching, which may reflect improved visualization and precision given by the robotic 3D screen and better ergonomics, although the clinical relevance of this difference seems to be minimal.

Unlike previous literature that focused almost exclusively on a single robotic system, our study is among the first to investigate the clinical performance of a multi-arm platform in urogynecological surgery on a high number of patients. The absence of significant differences in complication rates, functional outcomes, or patient satisfaction suggest that comparable outcomes may be achieved with different robotic systems; however, confirmation in multicentre settings is required.

Alternative explanations for the similar outcomes between groups may include the standardized surgical technique and high surgical expertise involved in both approaches. All procedures were performed by experienced surgeons with extensive training in both laparoscopic and robotic SCP, reducing variability related to proficiency.

Moreover, the uniform perioperative management protocols likely contributed to the comparable outcomes.

From a clinical perspective, the findings support the use of the Hugo™ RAS system as a valid option for SCP, especially in centres aiming to expand access to robotic surgery.

In an era where the robotic surgery market is rapidly expanding, emerging platforms have introduced competition and may address the cost barriers that have historically limited the use of robotic-assisted procedures in gynecology to oncologic or high-complexity cases [21, 22].

Most importantly, the comparable safety and efficacy profile allows clinicians to plan each surgery based on factors such as platform availability, patient preference, and clinical characteristics, rather than concerns about differential outcomes.

The study’s limitations include its retrospective, single-centre design and the relatively short follow-up period. All procedures were performed by only two experienced surgeons which may reduce the generalizability and may be reflective of outcomes.

While PSM reduced selection bias, unmeasured confounders may still exist. Long-term data on mesh-related complications and functional outcomes beyond the median follow-up of 18 months were not assessed in this study and will be investigated further. Moreover, the slightly longer operative time observed with Hugo RAS may decrease as institutional experience grows.

This study suggests that SCP performed with the Hugo™ RAS system offers comparable outcomes to traditional laparoscopy, without compromising safety or efficacy. For clinicians, this reinforces the role of RAS in urogynecological surgery and supports the integration of newer robotic platforms into clinical practice.

## Conclusions

In this propensity score matched cohort study, SCP performed using the Hugo™ RAS system demonstrated comparable safety, feasibility, and clinical effectiveness to conventional laparoscopy.

Both approaches had low complication rates, similar postoperative outcomes, and high patient satisfaction, with no significant differences in perioperative outcomes. While operative time was slightly longer with the robotic approach, this did not translate into adverse clinical outcomes and aligned with existing literature.

Our findings support the Hugo™ RAS system as a valid and effective alternative for minimally invasive SCP. Further multicentre and long-term studies are needed to confirm these findings and assess cost-effectiveness, learning curves, and durability of outcomes over time.

## Data Availability

Data is available from the authors upon reasonable request.
